# CD39 is expressed by a wide range of cutaneous T‐cell lymphomas

**DOI:** 10.1002/ski2.334

**Published:** 2024-01-10

**Authors:** Gilles Battesti, Nicolas Thonnart, Alizée Bozonnat, Caroline Ram‐Wolff, Adèle de Masson, Armand Bensussan, Martine Bagot, Anne Marie‐Cardine, Maxime Battistella

**Affiliations:** ^1^ Team 1 Human Immunology, Pathophysiology, Immunotherapy INSERM U976 Paris France; ^2^ Université Paris Cité IRSL Paris France; ^3^ Department of Dermatology Saint‐Louis Hospital Paris France; ^4^ Department of Pathology Saint‐Louis Hospital Paris France

## Abstract

CD39, an ectoenzyme in the immunosuppressive CD39/CD73/adenosine pathway, known to promote solid tumour outgrowth and spreading, was investigated in both skin and blood compartments of cutaneous T cell lymphomas. CD39 was overexpressed by peripheral blood T‐cells in Sezary syndrome and mycosis fungoides, and in skin‐infiltrating lymphocytes of Sezary syndrome, mycosis fungoides, subcutaneous panniculitis‐like T‐cell lymphoma and primary cutaneous CD30‐positive lymphoproliferation. Our study emphasizes the interest in using CD39/CD73/adenosine pathway blocking agents for cutaneous T cell lymphomas treatment.

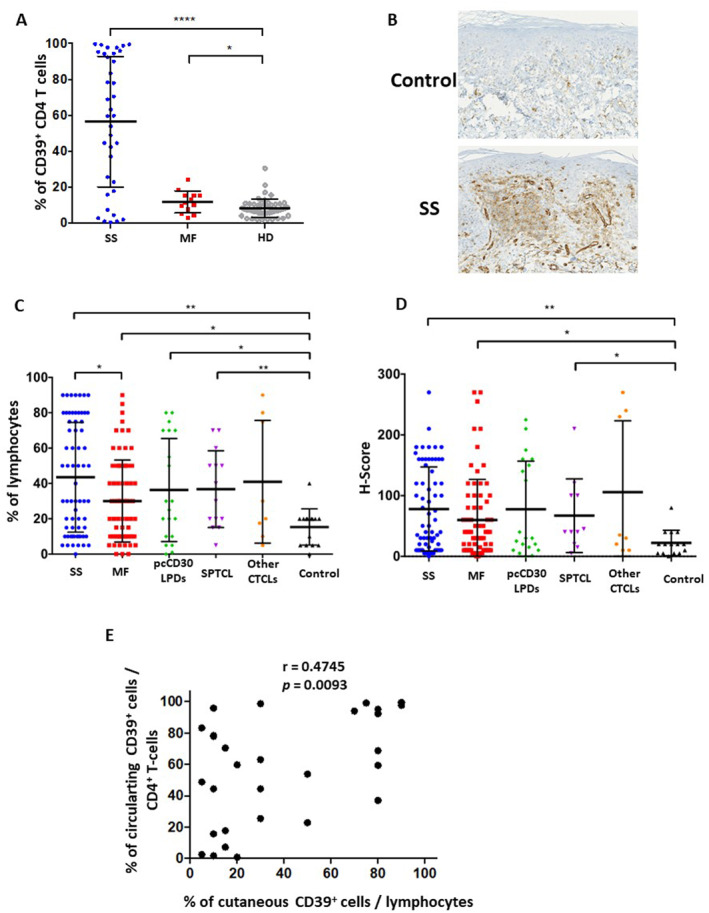

1

Dear Editor,

1

Cutaneous T‐cell lymphomas (CTCLs) are a heterogeneous group of diseases characterized by a tumour T‐cell clone originating from the skin. The two main forms are mycosis fungoides (MF) and leukaemic Sézary syndrome (SS) that, given the current state of therapeutic alternatives, present a poor prognosis at advanced stages.^1^

CD39 is an ectoenzyme involved in the CD39/CD73/adenosine pathway responsible for the generation of extracellular adenosine (eAdo) from adenosine triphosphate (ATP). eAdo was described as accumulating in solid tumours where it provides a broad immunosuppressive signal ultimately leading to the inhibition of antitumour immune responses, and consequently to tumour outgrowth and spreading.^2^ Accordingly, inhibition of the CD39/CD73/adenosine pathway with eAdo receptor antagonists, anti‐CD39 or ‐CD73 antibodies has demonstrated promising efficacy in the treatment of cancer.^3^, ^4^ We previously reported that CD39 was overexpressed by both malignant and non‐malignant peripheral blood T‐cells in SS patients, leading to an ATP‐mediated anti‐proliferative effect on non‐malignant T cells that was abolished in the presence of CD39/CD73/adenosine pathway inhibitors.^5^, ^6^ Similarly, Yakymiv et al. reported a CD39/CD73 dysregulation contributing to T‐cell immunosuppression in SS patients.^7^, ^8^ Targeting this pathway might therefore be of interest for developing new CTCL treatments. However, the T cell phenotypic heterogeneity between CTCL subtypes,^9^ exemplified in SS,^10^ could hinder such approach. To better explore the potential use of adenosine pathway's antagonists for promoting malignant cells clearance in both blood and skin compartments, we further investigated CD39 expression in the cutaneous compartment of various CTCL subtypes.

After patients' informed written consent, blood or/and skin samples from CTCL patients diagnosed according to the latest WHO‐European Organization for Research and Treatment of Cancer classification were collected. Blood from healthy donors (HD) and skin biopsies from patients with benign inflammatory skin disorders (ISD) were used as control samples. CD39 expression on peripheral blood T cells was assessed by multicolour flow cytometry analysis and results expressed as the percentage of CD39‐positive cells (PPc) among total CD4^+^ T cells. Cutaneous expression of CD39 was detected by immunohistochemistry (IHC) on paraffin‐embedded skin sections using anti‐CD39 antibody and scored to quantify PPc within total lymphocytes (range: 0–100), staining intensity (range: 0–3) and H‐score (=PPc × staining intensity; range: 0–300). The cutaneous lymphocytes were identified according to their morphological aspect.

As previously described,^5^ an overexpression of CD39 by peripheral blood CD4^+^ T cells was observed in SS (*n* = 37) and to a lesser extent in MF (*n* = 13) patients when compared to healthy individuals (HD, *n* = 47; Figure 1a). Similar results were obtained on lesional skin samples from SS (*n* = 66) and MF (*n* = 71) patients (patients' clinico‐biological data summarized in Table 1), by comparison with an age and sex‐matched ISD patients' control group (*n* = 15; representative labelling in Figure 1b). Cutaneous CD39 overexpression was confirmed by both PPc and H‐score values (Figure 1c,d). In MF and SS groups, no significant association (PPc and H‐score) was found between CD39 expression and the existence of a cutaneous large‐cell transformation, the TNMB stage, or the treated/untreated patient status at the time of skin sampling. For 29 SS patients, both cutaneous and blood samples were available (median time between samples = 4.5 months [range: 0–55.2]) which analysis revealed a moderate but positive correlation of CD39 expression between circulating CD4^+^ T cells and cutaneous lymphocytes (Figure 1e). Beside MF and SS, increased cutaneous CD39 expression was also detected in subcutaneous panniculitis‐like T cell lymphoma (SPTCL; *n* = 14), while primary cutaneous CD30‐positive lymphoproliferative disorders (pcCD30LPD; *n* = 20, including 15 cutaneous anaplastic large‐cell lymphomas and 5 lymphomatoid papulosis) displayed higher PPc than ISD control group, but no difference in H‐score (Figure 1c,d). Finally, no significant difference in CD39 expression was noticed between a group of rare CTCL (*n* = 8, including two primary cutaneous gamma‐delta T cell lymphomas, three extranodal natural killer/T cell lymphomas and three primary cutaneous peripheral T cell lymphoma, no other specification) and the ISD control group.

**FIGURE 1:**
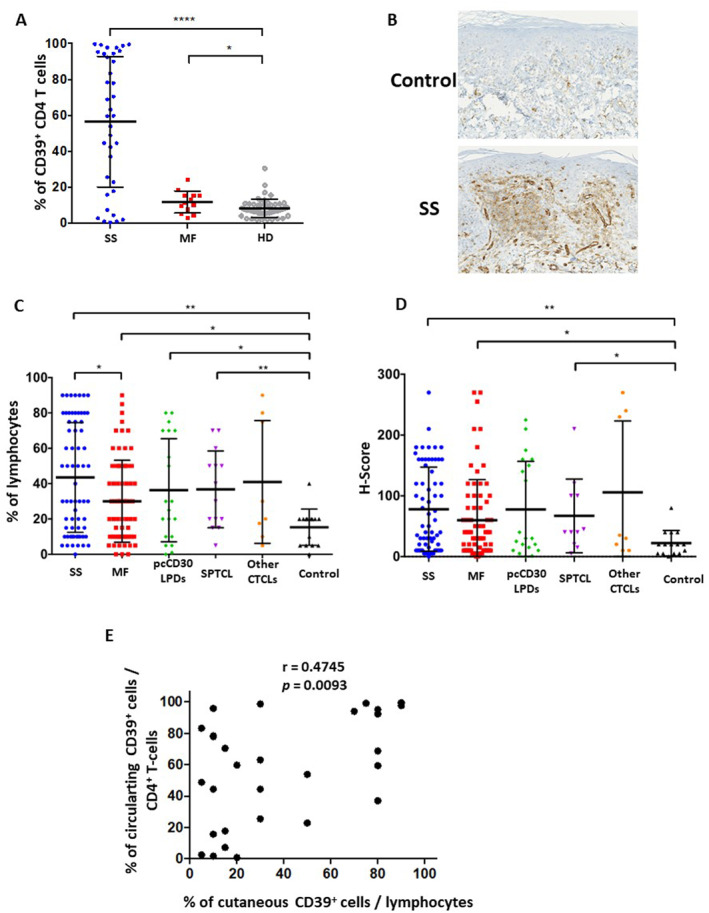
CD39 expression by peripheral blood and cutaneous T cells in CTCL. (a) Percentage of CD39^+^ cells within total CD4^+^ T cells evaluated by flow cytometry on blood samples of SS (*n* = 36), MF (*n* = 13) and HD (*n* = 47). (b–d) Expression of CD39 detected by IHC on skin sections from SS (*n* = 66), MF (*n* = 71), pcCD30LPDs (*n* = 20), SPTCL (*n* = 14), other CTCL subtypes (*n* = 8) and ISD (*n* = 15). (b) CD39 immunostaining on ISD and SS patient (×200 magnification). Quantification of (c) percentage of CD39^+^ cells among cutaneous lymphocytes (identified by their morphological aspect) and (d) H‐score. Statistical analyses performed using a Mann–Whitney *t* test. *p < 0.05, **p < 0.01, ****p < 0.0001. (e) Pearson correlation between CD39 expression by blood CD4^+^ T cells and cutaneous lymphocytes in SS (*n* = 29). CTCL, cutaneous T‐cell lymphoma; HD, healthy donors; IHC, immunohistochemistry; ISD, inflammatory skin disorder; MF, mycosis fungoides; SPTCL, subcutaneous panniculitis‐like T cell lymphoma; SS, Sézary syndrome.

**TABLE 1:** Clinico‐biological findings of MF and SS patients with studied lesional skin sample.

	MF *n* = 71	SS *n* = 66
Mean age (SD)	57.3 (23.4)	69.6 (37.4)
Female, *n* (%)	23 (32.4)	24 (40.0)
Time of sampling of the studied skin biopsy^b^	*n* = 59	*n* = 64
At diagnosis, *n* (%)	30 (50.8)	26 (40.6)
Under treatment, *n* (%)	29 (49.2)	38 (59.4)
Stage at skin biopsy time^a^ ^,^ ^c^		
T classification	*n* = 53	*n* = 63
T1, *n* (%)	13 (24.5)	3 (4.8)
T2, *n* (%)	25 (47.2)	13 (20.6)
T3, *n* (%)	9 (17.0)	6 (9.5)
T4, *n* (%)	6 (11.3)	41 (65.3)
N classification		
N0, *n* (%)	38 (71.7)	27 (42.9)
N1, *n* (%)	1 (1.9)	1 (1.6)
N2, *n* (%)	2 (3.8)	7 (11.1)
N3, *n* (%)	6 (11.3)	13 (20.6)
Nx, *n* (%)	3 (5.7)	15 (23.8)
M classification		
M0, *n* (%)	52 (98.1)	63 (100)
M1, *n* (%)	1 (1.9)	0 (0)
B classification^b^		
B0, *n* (%)	50 (94.3)	10 (15.9)
B1, *n* (%)	3 (5.7)	11 (17.5)
B2, *n* (%)	0 (0)	42 (66.7)
Stage		
IA, *n* (%)	12 (22.6)	1 (1.6)
IB, *n* (%)	17 (32.1)	5 (7.9)
IIA, *n* (%)	3 (5.7)	2 (3.2)
IIB, *n* (%)	8 (15.1)	2 (3.2)
IIIA, *n* (%)	6 (11.3)	2 (3.2)
IIIB, *n* (%)	0 (0)	4 (6.3)
IVA1, *n* (%)	0 (0)	34 (54.0)
IVA2, *n* (%)	6 (11.3)	13 (20.6)
IVB, *n* (%)	1 (1.9)	0 (0)
<IIB, *n* (%)	32 (60.4)	8 (12.7)
>IIA, *n* (%)	21 (39.6)	55 (87.3)

Abbreviations: MF, mycosis fungoides; SS, Sézary syndrome.

^a^ Using European Organization for Research and Treatment of Cancer‐Cutaneous Lymphoma Tumour Group updated blood classification.

^b^ The time of care (at diagnosis/under treatment) during which the biopsy was performed was unknown for 12 patients with MF and 2 with SS.

^c^Stage at skin biopsy time was unavailable for 18 patients with MF and 2 with SS.

Altogether our results identify CD39 as a marker for the cutaneous lymphocytes involved in CTCL, especially in SS, MF, SPTCL and pcCD30LPD subtypes. In addition, beside confirming our previous data showing CD39 overexpression by malignant and non‐malignant peripheral blood T cells in SS,^6^ they extend this observation to MF in the absence of blood tumour involvement. It therefore seems that CD39 bias of expression by circulating non‐malignant T cells could be indirectly induced by the presence of tumour cells in the skin compartment. In SS, CD39 expression appears correlated in the blood and skin compartments. However, this correlation could be misestimated due to delays between skin and blood sampling, or to the difference in CD39^+^ cells quantification inherent to the techniques used (evaluated by flow cytometry on circulating CD4^+^ T cells and by IHC on skin morphologically‐suggested lymphocytes). Nevertheless, our study emphasizes the interest in using CD39/CD73/adenosine pathway blocking agents for CTCL treatment.

## CONFLICT OF INTEREST STATEMENT

The authors declare no conflicts of interest.

## AUTHOR CONTRIBUTIONS

**Gilles Battesti**: Conceptualization (equal); data curation (equal); formal analysis (equal); methodology (equal); resources (equal); writing – original draft (equal); writing – review & editing (equal). **Nicolas Thonnart**: Data curation (equal). **Alizée Bozonnat**: Data curation (equal); investigation (equal); writing – review & editing (equal). **Caroline Ram‐Wolff**: Data curation (equal); methodology (equal). **Adèle de Masson**: Data curation (equal); investigation (equal); writing – review & editing (equal). **Armand Bensussan**: Data curation (equal); investigation (equal); writing – review & editing (equal). **Martine Bagot**: Investigation (equal); methodology (equal); writing – review & editing (equal). **Anne Marie‐Cardine**: Conceptualization (equal); data curation (equal); investigation (equal); methodology (equal); supervision (equal); validation (equal); writing – original draft (equal); writing – review & editing (equal). **Maxime Battistella**: Conceptualization (equal); data curation (equal); investigation (equal); methodology (equal); supervision (equal); validation (equal); writing – original draft (equal); writing – review & editing (equal).

## FUNDING INFORMATION

French Society for Dermatology, National Institute of Health and Medical Research (INSERM), University Paris Cité

## ETHICS STATEMENT

This study received the agreement of the local ethics committee (CPP 2019‐A01158‐49) and was conducted in accordance with the principles of the Helsinki declaration.

## Data Availability

The data underlying this article will be shared on reasonable request to the corresponding author.
